# The effects of family planning and other factors on fertility, abortion, miscarriage, and stillbirths in the Spectrum model

**DOI:** 10.1186/s12889-017-4740-7

**Published:** 2017-11-07

**Authors:** John Stover, William Winfrey

**Affiliations:** Avenir Health, Glastonbury, CT USA

**Keywords:** Spectrum, Avenir health, Lives saved tool, Family planning

## Abstract

**Background:**

The Lives Saved Tool (LiST) estimates the effects of maternal and child health interventions on mortality rates and the number of deaths. The family planning module in Spectrum interacts with LiST by providing estimates of the effects of scaling up family planning use on the number of live births, miscarriages, abortions, and stillbirths.

**Methods:**

We use the proximate determinants of fertility framework to estimate the effects of changes in contraceptive use, proportion married, postpartum insusceptibility, abortion and sterility on the total fertility rate. We extend this framework to estimate the number of intended and unintended pregnancies and the resulting live births, abortions, stillbirths, and miscarriages.

**Results:**

We apply the model to four countries (Mali, Kenya, Indonesia, and Ukraine) to demonstrate possible trends with a range of family planning and fertility levels. In high-fertility countries, such as Mali, increases in contraceptive use will partially compensate for the increasing number of women of reproductive age to reduce the annual increases in pregnancies and births. Most unintended pregnancies occur to women defined as having unmet need for contraception. In low-fertility countries, increases in contraceptive use may reduce abortion rates and low levels of unmet need mean that most unintended pregnancies are due to method failure.

**Conclusions:**

The family planning module in Spectrum provides a useful framework to incorporate changes in contraceptive practices and pregnancy outcomes in the LiST calculations of mortality rates and deaths.

## Background

**Fig. 1:**
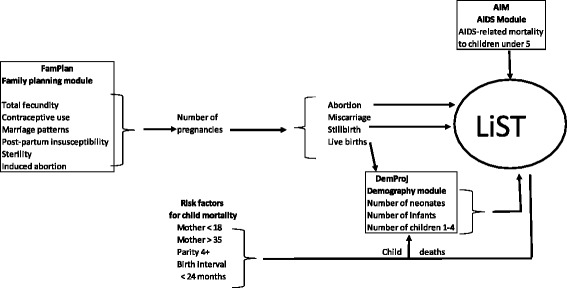
Structure of family planning effects in Spectrum

The Lives Saved Tool (LiST) model is concerned with rates of mortality for neonates, infants, children under five and mothers and the effects of health intervention scale-up on these rates. LiST also produces estimates of the total number of deaths and these are dependent on the number of live births, abortions, and stillbirths. LiST is a part of the Spectrum software package, which has other modules that provide information to LiST. The HIV module (AIM) estimates the effects of HIV interventions (anti-retroviral therapy, cotrimoxazole, and programs to prevent mother-to-child transmission of HIV) on child deaths due to AIDS. The demographic projection module (DemProj) estimates the number of live births, the number of children by single age and the number of deaths to children under 5 [1]. The family planning module (FamPlan) estimates the effects of contraception and other factors on the number of pregnancies and pregnancy outcomes. The resulting fertility rates are used by DemProj to calculate live births, and the number of abortions and stillbirths are used directly by LiST to estimate changes in the number of maternal and child deaths (Fig. 1).

Several frameworks have been proposed for analyzing the factors that determine human fertility. Hobcraft and Little developed an approach that examines the individual-level factors that affect individual fertility [2]. Wood developed a dynamic model of the proximate determinants of natural fertility (excluding contraception and induced abortion) that is also based on individual data [3].

An aggregate approach was proposed by Davis and Blake [4]. That approach recognized both indirect and direct determinants of fertility. Bongaarts developed these ideas into a useful framework for analyzing the proximate determinants of fertility [5–7]. His approach explains the fertility-inhibiting effects of the key direct determinants. The framework has been used for a variety of purposes, including (i) analyzing the contribution of changes in the proximate determinants to changes in the total fertility rate over time; (ii) comparing the differences in fertility between two countries or regions on the basis of differences in the proximate determinants; and (iii) estimating total abortion rates as a residual after the effects of all other proximate determinants have been removed.

Although there have been some criticisms of Bongaarts’ approach [3, 8] and suggested improvements to it [9], his framework is widely used for analyzing fertility and fertility change. It is the basis for the family planning calculations in Spectrum.

In this paper we first describe the methods used to relate pregnancy outcomes to measures of family planning need and use and then apply to model to four countries to illustrate the results in different settings.

## Methods

The proximate determinants of fertility framework developed by Bongaarts describes the factors that determine the observed total fertility rate (TFR): (i) marriage, (ii) contraception, (iii) postpartum insusceptibility, (iv) induced abortion, (v) sterility, and (vi) total fecundity. Definitions of these terms are provided below. The first five factors act to produce an observed TFR that is lower than total fecundity (the average number of births a woman would have if no factors were acting to inhibit fertility), as described in the following equation:

$$ {\mathrm{TFR}}_{\mathrm{t}}={\mathrm{Cm}}_{\mathrm{t}}\ \mathrm{x}\ {\mathrm{Ci}}_{\mathrm{t}}\ \mathrm{x}\ {\mathrm{Ca}}_{\mathrm{t}}\ \mathrm{x}\ {\mathrm{Cs}}_{\mathrm{t}}\ \mathrm{x}\ {\mathrm{Cc}}_{\mathrm{t}}\ \mathrm{x}\ \mathrm{TF} $$

where:

t = subscript denoting time

TFR = total fertility rate

Cm = marriage index

Ci = postpartum insusceptibility index

Ca = abortion index

Cs = sterility index

Cc = contraception index

TF = total fecundity

Definitions of these terms are as follows:

Cm = proportion married or in union

The index of marriage adjusts fertility for the proportion of the time from age 15 to 49 that a woman is sexually active. The index is simply the proportion of women of reproductive age who are married. Note that this implies that pregnancy does not occur to women outside of marriage. In countries where there is a considerable amount of pregnancy outside of marriage this term can be redefined as ‘in union’ to include informal relationships. In some cases, it may be appropriate to define this term as ‘sexually active’. In that case contraceptive prevalence needs to be among sexually active women rather than the standard definition of prevalence among married women.

$$ \mathrm{Ci}=20/\left(18.5,+,\kern0.025em ,\mathrm{PPI}\right), where\  PPI\  is the median duration of postpartum insusceptibility in months $$

The index of postpartum insusceptibility adjusts fertility for the period after a birth during which a woman is protected from pregnancy either due to the fertility-inhibiting effects of breastfeeding or postpartum abstinence. It is calculated as the ratio of the average birth interval in the absence of breastfeeding or postpartum abstinence (which Bongaarts estimated at 20 months) to the duration when these factors are taken into account, which is estimated as 18.5 months plus the median duration of postpartum insusceptibility.

$$ \mathrm{Cs}=\left(7.63\hbox{--} 0.11\ \mathrm{x}\ \mathrm{s}\right)/7.3, where\ s\  is the percentage of women aged\  45- 49\  who\  have\  had\  no\  live births $$

The index of sterility is estimated from a regression equation that uses the prevalence of primary sterility (the proportion of women who cannot get pregnant due to biological factors) to estimate the effects of primary and secondary sterility on fertility. Secondary sterility occurs when women of reproductive age who have had one or more births can no longer conceive. This index may be over-estimated in cases of voluntary childlessness.

Ca = TFR / (TFR + (0.4 x (1 + CPR) x TAR), *where TFR is the total fertility rate, CPR is the contraceptive prevalence rate, and TAR is the total abortion rate (the average number of abortions per woman during her lifetime)*

The index of abortion adjusts fertility for those pregnancies that are terminated by abortion. Each abortion reduces total fertility by less than one birth because it results in a shorter period of gestation and more rapid return of fertility than a full term pregnancy. The net effect is estimated to be 0.4 births averted per abortion in the absence of contraceptive use. Higher rates of contraceptive use lead to larger effects of abortion on fertility since the risk of conception is lower than without contraception. Since the index of abortion is used to calculate the total fertility rate this equation is usually applied using TFR from the previous year.

$$ \mathrm{Cc}=1\hbox{--} 1.08\ \mathrm{x}\ \mathrm{CPR}\ \mathrm{x}\ \mathrm{e} $$

The index of contraception expresses the fertility inhibiting effects of contraception as a function of the proportion of women using contraception (CPR) and the average effectiveness of contraception given the method mix (*e*). The term 1.08 in the equation adjusts for that fact that some women who are sterilized may be post-menopausal, in which case the use of contraception has no fertility effect. Average effectiveness is an average effectiveness of each method weighted by the proportion of users using that method. Standard method failure rates are based on analysis of data from developing countries by Cleland [10] and the United States by Trussel [11], as shown in Table 1.

**Table 1:** Contraceptive failure rates by method

Method	Failure rate	Source
Female sterilization	0.50%	Trussel
Male sterilization	0.15%	Trussel
Oral pill	6.90%	Cleland
IUD	1.60%	Cleland
Injections	2.90%	Cleland
Implants	0.05%	Trussel
Male condom	9.80%	Cleland
Lactational amenorrhea	24.0%	Trussel
Traditional methods	78.0%	Trussel

*Note:* The failure rate is defined as the proportion of women using a method who will become pregnant in a year due to failure of the method

Total fecundity (TF) is the total number of live births women would have if none of these proximate determinants were acting to reduce her fertility; if she were continually married from age 15 to 49, did not breastfeed, did not experience primary or secondary sterility, did not have an abortion, and did not use family planning.

The value of total fecundity is unknown and appears to vary across countries due to factors not included in this framework (nutritional status, spousal separation, frequency of intercourse, etc.). Bongaarts suggested the range should be from about 13 to 18. Total fecundity can be estimated for any year in which data on TFR and the other proximate determinants are available, usually from a national household survey. The proximate determinants equation can be re-arranged to estimate total fecundity in that year, as follows:

$$ \mathrm{TF}=\mathrm{TFR}/\left(\mathrm{Cm}\ \mathrm{x}\ \mathrm{Ci}\ \mathrm{x}\ \mathrm{Cs}\ \mathrm{x}\ \mathrm{Ca}\ \mathrm{x}\ \mathrm{Cc}\right) $$

We assume that total fecundity is constant over time. Thus, once the value of total fecundity is determined, the proximate determinants equation can be used to calculate the effects of changes in any of the proximate determinants on the total fertility rate. Using this framework, the family planning module in Spectrum calculates the TFR that results from future changes in contraceptive use, method mix, and the other proximate determinants.

There are some limitations to this framework. The major limitation is that it estimates the total fertility rate, which can be used to estimate live births, but tells us nothing about pregnancies, pregnancy intentions, or pregnancy outcomes. In the FamPlan modue of Spectrum, we extend the framework to estimate the number of pregnancies from live births by adding miscarriages, stillbirths, and abortions.

Miscarriage (or spontaneous abortion) refers to natural pregnancy losses early in a pregnancy, usually before 20 weeks of pregnancy. The rates vary widely depending on how they are measured (from conception, at 4 weeks, at 8 weeks, etc.). By default, we use a miscarriage rate of 13% as estimated by Bongaarts and Potter [12].

Stillbirths are pregnancy losses later in pregnancy, usually as 20 or 28 weeks of pregnancy. Country-specific estimates of stillbirth rates are available and indicate a global average of about 19 stillbirths per 1000 live births in 2009 [13].

Induced abortion refers to a procedure to terminate a pregnancy. The majority are done in the first 8 weeks of pregnancy and almost all are done before the 13th week. Abortions in the second trimester (13 weeks or later) or third trimester are generally rare. Rates of induced abortion are difficult to measure, in part because induced abortion is illegal in many countries. Estimates of induced abortion rates are available [14] and suggest that worldwide about 25% of pregnancies end in induced abortion, with a variation from about 13% in Middle Africa to 39% in the Caribbean. An induced abortion is a response to an unwanted pregnancy. Some unintended pregnancies are unwanted but not all. Also the rates of unintended pregnancies will change as contraceptive use changes. Therefore, it is preferable to express the induced abortion rate as a proportion of unintended pregnancies.

It is difficult to estimate the proportion of pregnancies that are unwanted, partially because a woman may change her mind once she becomes pregnant. However, we can estimate the number of unintended pregnancies as those resulting from two sources: method failure and pregnancies occurring to women with an unmet need for contraception. Pregnancies due to method failure are calculated using the method failure rates given in Table 1. The annual pregnancy rate among women with an unmet need for contraception is estimated to be 31% (inter-quartile plausibility range of 23–38%) [15].

**Fig. 2:**
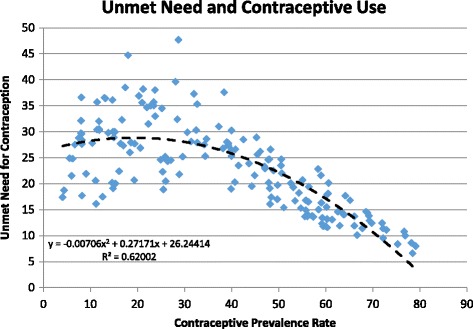
Relationship between unmet need for contraception and contraceptive prevalence rate. Source: DHS data for 169 surveys from 71 countries [17]

Unmet need is defined as the proportion of fecund women who are not using contraception (modern or traditional) and are: (i) at risk of becoming pregnant but do not want to become pregnant in the next 2 years or ever or are unsure of their pregnancy intentions; (ii) pregnant with a mistimed or unwanted pregnancy; or (iii) postpartum amenorrheic for up to 2 years following a birth that was mistimed or unwanted [16]. Unmet need is measured in national household surveys and represents a rough estimate of the degree to which demand for contraceptive use exceeds actual use. Levels of unmet need vary as the use of contraception changes. The average pattern is shown in Fig. 2. Unmet need is typically low when use of family planning is very low. Demand tends to increase faster than use as CPR rises from zero to about 20% leading to increased unmet need. Then contraceptive use rises faster than demand and the proportion of women with unmet need declines.

With this approach, we can assume that the proportion of unintended pregnancies terminated by induced abortion remains constant over time, while the actual abortion rate will vary in accordance with changes in contraceptive use and unmet need. Note that this approach does not account for abortions that are done for the purposes of sex selection.

Thus we have the following equations to estimate the number of pregnancies and birth outcomes:

$$ \mathrm{P}=\mathrm{B}+\mathrm{A}+\mathrm{M}+\mathrm{S} $$

$$ \mathrm{A}=\mathrm{U}\ \upalpha $$

$$ \mathrm{U}=\mathrm{CU}\ \mathrm{x}\ \left(1\hbox{--} {\mathrm{e}}_{\mathrm{t}}\right)+\mathrm{UN}\ \mathrm{x}\ \uprho $$

$$ {\mathrm{M}}_{\mathrm{t}}=\left(\mathrm{B}+\mathrm{A}+\mathrm{S}\right)\ \mathrm{x}\ \upmu /\left(1-\upmu \right) $$

$$ \mathrm{S}=\mathrm{B}/1000\ \mathrm{x}\ \upsigma $$

Where

P = number of pregnancies

B = number of live births

A = number of induced abortions

M = number of miscarriages

S = number of stillbirths

U = number of unintended pregnancies

CU = number of women using contraception

UN = number of women with unmet need for contraception

α = proportion of unintended pregnancies terminated by abortion

μ = proportion of pregnancies ending in miscarriage

σ = stillbirths per 1000 live births

ρ = pregnancy rate for women with unmet need

These calculations are implemented in the family planning module in Spectrum and produce estimates of the total fertility rate, pregnancies, abortions, and stillbirths. The demographic module estimates the number of live births, and LiST uses all this information to estimate maternal and child mortality, stillbirths, and the effects of family planning on mortality. Note that since maternal mortality rates are different for live births, stillbirths, and induced abortions, the changing distribution of pregnancy outcomes can change the maternal mortality ratio as well as the number of maternal deaths.

**Fig. 3:**
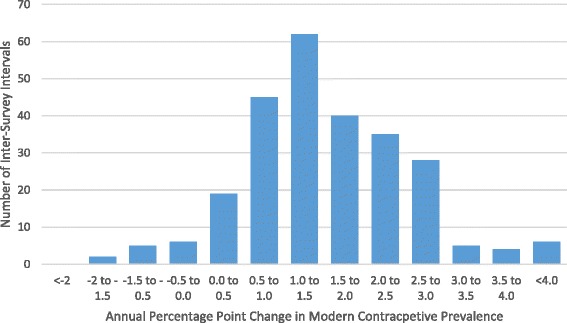
Distribution of the annual rate of change of modern contraceptive prevalence. Source: DHS data for 257 inter-survey intervals from 93 countries [17]

The effect of an increase in contraceptive use on number of pregnancies and pregnancy outcomes depends on the magnitude of scale-up. While the model can provide estimates for any increase in contraceptive use, for planning purposes it is useful to use realistic rates of scale-up. Figure 3 shows the annual percentage-point change in modern contraceptive prevalence for 257 inter-survey intervals for 93 countries with multiple Demographic and Health Surveys [17]. Each point represents the annual increase between two consecutive surveys. The average increase is about 1 percentage point per year. About 30% of the intervals have a growth rate above 2 points per year and only 6% have more than 3 points per year.

## Results

In order to demonstrate the implications of the proximate determinates framework for a model such as the Spectrum software package, we will illustrate the range of effects of family planning on the number of pregnancies and pregnancy outcomes by examining four countries:

- Mali, as an example of a country with low use of contraception and high fertility
- Kenya, as an example of a country with moderate use of contraception and medium fertility
- Indonesia, as an example of a country with high use of contraception and low fertility
- Ukraine, as an example of a country with moderate use of modern contraception but very low fertility due to the use of traditional methods and abortion

The values of selected key indicators for these four countries are shown in Table 2. Modern contraception includes sterilization, IUD, oral pills, injections, implants, condoms, and lactational amenorrhea. Traditional methods include withdrawal and periodic abstinence. Mali has high fertility, low contraceptive use, high unmet need, high proportion married, and long periods of postpartum insusceptibility. The long period of postpartum insusceptibility is due primarily extended breastfeeding; the median duration is 11.7 months. The lower levels of fertility in the other countries are associated with higher levels of contraceptive use and lower levels of unmet need, proportion married, and shorter duration of postpartum insusceptibility. As stated above, the high rate of induced abortion is a major contributor to low fertility in Ukraine. These characteristics imply that in Mali about one-third of pregnancies are unintended and most of those are due to unmet need for contraception. By contrast, in Ukraine, over 55% of pregnancies are unintended and most of those are due to method failure.

**Table 2:** Key reproductive indicators for four countries

	Mali	Kenya	Indonesia	Ukraine	Ref.
Total fertility rate	6.1	3.9	2.6	1.2	[18–21]
Modern contraceptive prevalence (among married women)	10%	53%	58%	48%	[18–21]
Proportion of married women using traditional methods	1%	5%	4%	19%	[18 = 21]
Unmet need	26%	18%	11%	10%	[18–21]
Percent of women 15–49 married or in union	85%	60%	73%	60%	[18–21]
Duration of postpartum insusceptibility (months)	11.7	8.6	3.8	3.9	[18–21]
Percent of women 45–49 who are childless	2.2%	0.9%	2.4%	3.5%	[18–21]
Abortions per 1000 women	31	34	35	42	[14]
Percentage of pregnancies ending in abortion	12%	14%	27%	38%	[14]
Total fecundity	13.3	22.9	12.9	6.5	Calculated
Stillbirth rate	44	26	18	12	[13]
Average effectiveness of contraception	95%	96%	96%	94%	Calculated
Annual number of					
Pregnancies	970,000	2,200,000	7,900,000	860,000	Calculated
Unintended pregnancies	308,000	710,000	3,300,000	480,000	Calculated
Births	732,000	1,500,000	4,900,000	470,000	Calculated
Miscarriages	126,100	286,000	1,027,000	111,800	Calculated
Stillbirths	32,000	40,000	87,000	5800	Calculated
Abortions	110,000	340,000	1,900,000	280,000	Calculated

**Fig. 4:**
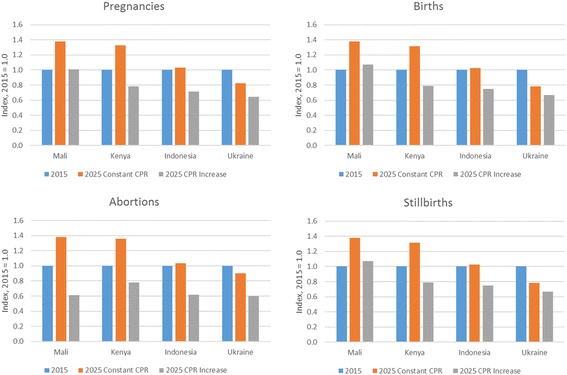
Change in the number of pregnancies, births, abortions, and stillbirths over 10 years under constant or increasing contraceptive use scenarios

The effects of increases in contraception can be illustrated by comparing two scenarios: first, assuming that contraceptive prevalence remains constant, and second, assuming that contraceptive prevalence increases by two points a year in Mali and Kenya and by one point per year in Indonesia and Ukraine, for a period of 10 years. This latter scenario results in an increase in contraceptive prevalence from 10% to 30% in Mali, from 42% to 62% in Kenya, from 62% to 72% in Indonesia, and from 67% to 77% in Ukraine. The effects of these increases on the pregnancies, births, abortions, and stillbirths can be seen in Fig. 4. Due to high rates of population growth in Mali and Kenya, the numbers of pregnancies and other outcomes would increase from 2015 to 2025 if contraceptive use were to remain constant. With an increase in contraceptive use, pregnancies and live births would remain roughly constant in Mali but fall in the other three countries. The number of abortions would fall in all four countries.

With constant use of contraception, the number of children under the age of five would increase by 33% from 2015 to 2025 in Mali and by 27% percent in Kenya. The increase would be only 1% in Indonesia, and Ukraine would experience a 19% decline because of its low fertility. With an increase in contraceptive use, the number of children under five would increase by only 8% in Mali and would decrease in Kenya, Indonesia, and Ukraine by 14%, 21%, and 29%, respectively. These changes will be reflected in the number of maternal and child deaths estimated in LiST, even if mortality rates remain constant.

## Discussion

Changes in the use of contraception can have important effects on maternal and child survival. An increase in contraceptive use leads to a reduction in the number of births which, all other things being equal, means fewer maternal deaths, fewer stillbirths, and fewer children exposed to the risk of mortality. An increase in contraceptive use may also affect the number of abortions, which affects maternal mortality. Changes in other proximate determinants – particularly marriage rates, abortion, and breastfeeding practices – can also have important effects on fertility.

The approach used in the Spectrum software package allows the effects of family planning and the other proximate determinants to be included in LiST calculations in a consistent framework that links contraceptive use, fertility desires, abortion practices, and demographic processes to the maternal and child mortality calculations in LiST.

There are several limitations to this approach. The proximate determinants concept represents a useful framework to capture the main effects of interest, but it does not fully explain all the factors affecting fertility. The large variation in estimated levels of total fecundity probably reflect variations in other characteristics that affect fertility but are not included in the framework and may be unmeasured. Unlike LiST, the family planning module does not simulate the effects of interventions designed to increase contraceptive use (such as postpartum family planning, social marketing, or community-based distributions programs) or the introduction of new methods, but instead requires the user to enter assumptions about future changes in contraceptive use. We are working to include these dynamics in future versions. Estimates of abortion and unmet need are subject to error and, therefore, estimates of the proportion of unintended pregnancies terminated by abortion are uncertain. We assume that these proportions remain constant with time but that may not be the case when medical options or the legal environment change.

Change in rates of contraceptive use are associated with changes in the distribution of births by key risk factors (short birth intervals, high parity, and maternal age below 18 or above 35) that are associated with elevated child mortality rates. We have examined these relationships previously in order to include them in the model, but research so far has found only weak causal effects [22–24].

The Sustainable Development Goals [25] call for improvements in many aspects of health, including family planning as well as maternal and child survival. The Spectrum software package provides a system to examine these effects jointly, in order to capture the synergies that can be important to estimating future trends in child survival.

## Conclusion

The number of child deaths in any population at any time results from a large number of factors that determine the number of births, the risks to which children are exposed and the health services they receive. The LiST model focuses on causes of death and the impact of interventions. The FamPlan and DemProj modules in Spectrum provide LiST with the number of live births each year as well as the number of stillbirths and abortions by considering the influences of contraceptive use, marriage and breastfeeding patterns and abortion rates. This link provides analysts and planners with a comprehensive picture of the factors that determine the number of child deaths.
